# Mitochondria in *Mycobacterium* Infection: From the Immune System to Mitochondrial Haplogroups

**DOI:** 10.3390/ijms23179511

**Published:** 2022-08-23

**Authors:** Felipe Gouvea de Souza, Giovanna C. Cavalcante

**Affiliations:** Laboratory of Human and Medical Genetics, Graduate Program in Genetics and Molecular Biology, Federal University of Pará, Belém 66075-110, Brazil

**Keywords:** mycobacteria, mitochondria, *M. leprae*, *M. tuberculosis*, haplogroups, immune response, bacterial infection

## Abstract

In humans, mitochondria play key roles in the regulation of cellular functions, such as the regulation of the innate immune response and are targets of several pathogenic viruses and bacteria. Mycobacteria are intracellular pathogens that infect cells important to the immune system of organisms and target mitochondria to meet their energy demands. In this review, we discuss the main mechanisms by which mitochondria regulate the innate immune response of humans to mycobacterial infection, especially those that cause tuberculosis and leprosy. Notably, the importance of mitochondrial haplogroups and ancestry studies for mycobacterial diseases is also discussed.

## 1. Introduction

Mitochondria participate in several processes in cellular function and operation through mechanisms such as mito-nuclear signaling, energy generation, storage and control of calcium levels, lipid homeostasis, and immune responses against infectious and parasitic agents [1,2,3]. It is noteworthy that this immune signaling is essential for defense mechanisms against mycobacteria, obligate intracellular pathogens that infect macrophages and other cells in organisms, causing a spectrum of clinical phenotypes [4,5,6].

Among the main mycobacterial agents that cause disease in humans, tuberculosis and leprosy stand out [7,8,9]. Tuberculosis is the leading cause of death from a single infectious agent worldwide, particularly in Africa, Southeast Asia, and the Western Pacific, as well as being the leading cause of death among people with HIV [10,11]. Leprosy, in this same perspective, is an endemic disease prevalent in tropical countries, with emphasis on underdeveloped and developing countries around the world [12,13].

It is worth mentioning that mycobacteria have developed a dependence on the energy production and nutritional products of the host, targeting the mitochondria, due to the cell signaling pathways in which this organelle participates and connects its metabolism to meet their nutrient demands [4,8,14].

Here, considering these are still understudied infections in this perspective, we discuss the mechanisms and mitochondrial pathways reported in the literature that are associated with the innate host immune response to mycobacterial infection, focusing on leprosy and tuberculosis, in addition to the importance of mitochondrial haplogroups and ancestry studies considering both the mitochondrial genome and the nuclear genome for mycobacterial diseases.

## 2. Mitochondria

Mitochondria are cytoplasmic organelles that participate in important processes in cellular function, including cell death, control of calcium levels, lipid homeostasis, metabolic cell signaling, and the generation of about 90% of cellular energy in the form of Adenosine Triphosphate (ATP), mainly by oxidative phosphorylation (OXPHOS), and by the tricarboxylic acid cycle (TCA) [1,2].

Structurally, mitochondria have a double membrane with distinct lipid and protein compositions between the outer and inner layers. The outer membrane is smooth, highly permeable, and only slightly selective for cytosolic solutes [3,15]. The inner membrane, on the other hand, is folded into structures called cristae, less permeable and highly selective for solutes, also carrying the system responsible for OXPHOS, known as the electron transport chain (ETC), which is composed of five protein complexes (Complexes I–V) [1,15,16]. In addition, the spaces generated by these membranes define two distinct compartments: the intermembrane space (IMS) and the mitochondrial matrix, where the TCA cycle occurs [3,15].

Due to their evolutionary origin, mitochondria have double-stranded circular DNA as their own genetic material (mtDNA), located in the mitochondrial matrix and associated with the inner membrane of the organelle [17,18]. Human mtDNA has 16,569 bp, comprising 37 genes, 13 of which code for OXPHOS-associated polypeptides, 22 are transfer RNA (tRNA) genes, and two are ribosomal RNA (rRNA) genes. Among the non-coding regions of mitochondria, there is the largest region, called the NCR, which encompasses sequences known as the displacement loop (D-loop), essential for replication and transcriptional regulation [1,2,15].

Protein-coding genes generate central subunits of ETC complexes I, III, IV, and V (the complex II subunits are not encoded by mtDNA), with seven of these genes encoding complex I subunits (*MT-ND1*, *MT-ND2*, *MT-ND3*, *MT-ND4L*, *MT-ND4*, *MT-ND5,* and *MT-ND6*), where OXPHOS mostly starts [15].

It is noteworthy that OXPHOS is an important step in energy production for the generation of reactive oxygen species (ROS) due to the high propensity to release free electrons by ETC [15,19]. While several different antioxidant proteins within mitochondria neutralize these molecules, excessive production of ROS can cause damage to mtDNA, considering ROS dysregulation of its genome influences the process of the inflammatory response against infectious pathogens [19].

It is also important to note that each cell has hundreds to thousands of copies of mtDNA molecules, and alteration of mtDNA content can be affected by both genetic and physiological environment factors, such as thyroid hormone level [4,19]. Evidence has shown that mtDNA copy number control is very important for mitochondrial biogenesis and normal cell function, and mtDNA copy number loss has been reported as the cause of mtDNA depletion syndrome, which may be also involved in complex diseases such as cancer, diabetes, and neurodegenerative and infectious diseases [4].

Another important regulatory factor of this organelle is mitochondrial dynamics, which is the change in mitochondrial number, structure, and positioning within the cytoplasm by regulatory processes of fusion and fission and by the formation of mitochondrial networks that modulate diverse biological functions, including mitochondrial metabolism, redox balance, and cell death [20,21,22].

Mitochondrial fusion is characterized by the union of two mitochondria, specifically the fusion of the outer mitochondrial membrane (OMM) and the inner mitochondrial membrane (IMM) into only one mitochondrion [20,23]. By this process, a partially damaged mitochondria can join other healthy mitochondria, leading to the exchange of metabolites, thus diluting the accumulated mutations in the mitochondrial DNA and being critical to sustaining mitochondrial functions [21]. Mitochondrial fusion is controlled by mitofusin-1 (MFN1) and mitofusin-2 (MFN2) GTPases, which are prerequisites for OMM fusion, and by optical atrophy GTPase 1 (OPA1), located in the IMM [21,22,23].

In this sense, MFN1 and MFN2, which are OMM proteins, interact with each other in a homo and heterotypic manner involving the hydrolysis of GTP, oligomerize around adjacent mitochondria, and eventually lead to OMM fusion [21,23]. Furthermore, MFN1 has a higher GTPase activity as well as a tighter mitochondrial binding capacity than MFN2; however, MFN2 is essential for mitochondria-associated membrane formation and other cellular functions [21].

IMM fusion is mediated by OPA1, which is a large dynamin-related GTPase protein anchored in the IMM [21,23]. OPA1 is proteolytically cleaved into different fragments by the OMA1 and YME1L proteins under normal conditions to produce a long form (L-OPA1) and a short form (S-OPA1), in which L-OPA1 is intrinsic in IMM and S-OPA1 is in the intermembrane space (IMS) [21,23].

Mitochondrial fission is characterized by the division of one mitochondrion into two daughter mitochondria and is necessary to remove damaged parts of the mitochondria, which are eventually eliminated by mitophagy, in addition to having a critical role in the replication of mitochondria during the cell cycle [20,21,23].

Mitochondrial fission is regulated by the dynamin-related protein 1 of the cytosolic GTPase protein (DRP1) through interaction with other OMM proteins such as MiD51 and MiD49, where it forms oligomeric structures surrounding the mitochondrial scission site and eventually, through GTP hydrolysis, undergoes a conformational change that results in membrane constriction and scission [22,23]. In addition, these proteins are essential for mitochondrial quality control and homeostasis, as well as the regulation of numerous biological processes, including immune responses [22,23].

## 3. Mitochondria in the Regulation of Innate Immune Response

Mitochondria play an essential role in several signaling and homeostasis pathways, in the regulation of intrinsic apoptosis and other types of cell death, and in cell cycle participation [2,3]. Among these metabolic pathways, mitochondria have also been highlighted as important regulators of the innate immune system in humans, so that dysregulation of mitochondrial functioning, including energy generation, can affect nucleus-mitochondria communication and the response against infections [24,25].

The innate immune defense system is effective in fighting the infection of different pathogens, being the first line of defense against these microorganisms [24]. Invading pathogens are recognized by a series of pattern recognition receptors (PRRs) that have evolved to detect a wide range of pathogen-associated molecular patterns (PAMPs), conserving features of invading organisms that serve to identify them as foreign, such as bacterial lipopolysaccharides (LPS) and viral nucleic acids [24,26,27].

In addition, this defense system also recognizes eukaryotic cell-derived molecules themselves, by damage-associated molecular patterns (DAMPs), that bind to specific receptors, including RIG-I-like receptors (RLRs), NOD-like receptors (NLRs), and Toll-like receptors (TLRs), to generate cytokines essential for the elimination of these organisms or in the repair of tissue damage [24,26,28].

**Figure 1:**
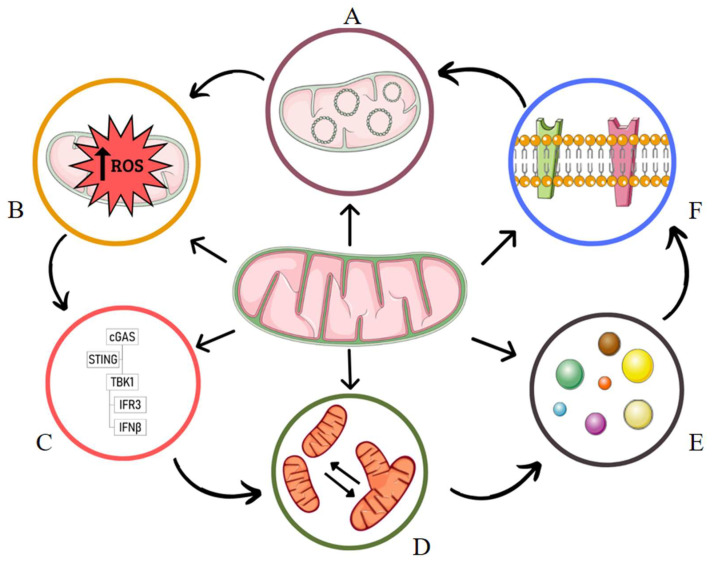
Mitochondrial pathways in the immune response. A: Mitochondrial DNA. B: ROS accumulation. C: cGAS-STING.D: Mitochondrial dynamics. E: Inflammassomes. F: TLR receptors.

Mitochondria have several potent immunostimulatory DAMPs, including their own genome, that activate the innate immune system upon exposure to the cytosol or release into the extracellular environment in response to cellular stress and loss of homeostasis [24,27]. Figure 1 illustrates mitochondrial pathways in the immune response.

### 3.1. Toll-like Receptors

Toll-like receptors (TLRs) are intracellular or membrane-bound PRRs that recognize a multitude of different bacterial traits to instigate innate immunity, being essential for eliminating pathogens or repairing tissue damage [26,28,29]. In humans, ten TLRs were identified, with TLRs 1, 2, 4, 5, 6, and 10 found on the cell surface and receptors 3, 7, 8, and 9 located on the endosomal membrane [27]. Activated by ligand binding to its carboxy-terminal leucine-rich repeat and expressed by all innate immune cells, which detect DAMPs or PAMPs [27,28].

In this sense, among the important functions that mitochondria are known to play in the innate immune response to microbial infection is their recognition by TLR9, an innate immune receptor capable of recognizing bacteria or viruses by binding to unmethylated CpG motifs in DNA [4,30]. As mitochondria share similarities with the bacterial genome, due to their evolution from alphaprotobacteria to endosymbionts and, finally, cytoplasmic organelles, the mitochondrial genome (mtDNA) contains CpG DNA repeats and codes for formylated peptides [31]. In this sense, TLR9 also recognizes cytoplasmic mtDNA when released into the plasma because of acute trauma and injury [4,30,32].

TLR9 is synthesized in the endoplasmic reticulum (ER) and is targeted to the endolysosome for DNA recognition, where its signaling proceeds through adapter myeloid differentiation primary response protein 88 (MYD88), which activates mitogen-activated protein kinases (MAPKs) and nuclear factors (e.g., NF-κB), triggering the expression of pro-inflammatory genes, or through interferon regulatory factor 7 (IRF7) to enhance IFN type I (IFN-I) responses in dendritic cells or other immune cells [27,33,34].

### 3.2. Inflammasomes

Another important mechanism by which mtDNA triggers an immune response is through interaction with inflammasomes, multisubunit cytoplasmic multiprotein complexes that modulate inflammatory responses through caspase-1 (CASP1) activation and secretion of pro-inflammatory cytokines IL-1β and IL-18 [30,33].

The main receptors capable of activating inflammasomes are the NLR family, of which NLRP3 stands out, which are activated by endogenous PAMPs and/or DAMPs released during necrosis, cellular stress or in the face of mitochondrial dysfunction [33,35,36].

In this regard, NLRP3 is expressed in macrophages, monocytes, dendritic cells, neutrophils, as well as numerous non-hematopoietic cells [27]. Upon stimulation, NLRP3 binds to apoptosis-associated speck-like protein (ASC) from its pyrin domain to induce recruitment of the pro-caspase-1 effector molecule through interactions of the caspase recruitment domain (CARD) to thereby form the NLRP3 inflammasome [34,37].

Inflammasome assembly triggers caspase-1 auto-cleavage and activation, converting pro-IL-1β and pro-IL-18 into their mature forms, which respond to highly diverse stimuli, including ATP, bacterial toxins, microcrystalline substances, lipid particles, bacteria, and viruses [35,36,37]. This activation has been linked to a wide range of infectious inflammatory disorders, including bacterial, viral, fungal infections, metabolic syndromes, atherosclerosis, and Alzheimer’s disease [27].

Production of mitochondrial reactive oxygen species (mtROS), release of mitochondrial DAMPs, and altered mitochondrial dynamics have been linked to inflammasome activation, although the exact mechanisms by which mitochondria engage NLRP3 and other inflammasomes remain under investigation [33]. One proposal is that through different mechanisms that may involve rupture of the plasma membrane, such as K+ efflux and intracellular Ca2+ increase, and NLRP3 activators cause a form of mitochondrial damage that causes the release of fragmented mtDNA and increased production of ROS that convert mtDNA to an oxidized form (ox-mtDNA), which supports mtDNA as an endogenous agonist of inflammasomes [33,35]. Furthermore, it was observed that inhibition of mitochondrial complex I by rotenone and of complex I by antimycin A induces robust production of ROS from mitochondria, which activates the NLRP3 inflammasome, highlighting the role of mtROS in the activation and regulation of NLRP3 [38].

### 3.3. cGAS-STING

Interferon gene stimulators (STING) are adapter molecules of great importance in the innate immune response, involved in the detection of cytosolic DNA, a potent activator of an IFN-I response, and cell activation, also detecting cyclic nucleotides, stimulating the induction of IFN-I [39,40].

Under normal conditions, DNA is confined to the nucleus and mitochondria and is rapidly degraded by nucleases in the cytosol and endolysosomal compartments [40]. Following infections, however, increased amounts of intracellular DNA are detected in a pathway that involves cyclic GMP–AMP synthase [40].

To regulate the induction of IFN-I in exogenous and endogenous DNA, the cGAS-STING signaling axis emerged. The cGAS enzyme detects cytoplasmic DNA for the formation of a dimer [29,33]. cGAS then undergoes a conformational change that facilitates the conversion of ATP and GTP to cyclic 2′3′-GMP-AMP (cGAMP), a second messenger that binds to the stimulator protein residing in the endoplasmic reticulum, and activates STING, inducing a conformational change in its C-terminal [29,33].

Consequently, TANK-binding kinase 1 (TBK1) is recruited to activated STING, that phosphorylates interferon regulatory factor 3 (IRF3) to promote its homodimerization and translocation to the nucleus, causing IFNβ expression and transcription of hundreds of interferon-stimulated genes (ISGs) that are potentially antiviral [29,33].

It is important to emphasize that TBK1 is an important regulator of innate immune responses to viruses or bacteria and function as key regulators of apoptosis, autophagy, and inflammatory responses and act as important inducers that drive tumorigenesis [41,42].

Regarding TBK1 signaling in mitochondria, TBK1 activation involves the two autophagy adapters OPTN and NDP52, cargo adapter proteins involved in selective autophagy, and the ability of OPTN to bind mitochondrial poly-UB chains in response to mitochondrial damage [43]. Furthermore, activation of TBK1 in response to mitochondrial depolarization promotes phosphorylation of charge adapter proteins such as SQSTM1, OPTN, and NDP52, and TBK1 activity is required for the efficient recruitment of OPTN, NDP52, and SQSTM1 to depolarized mitochondria [43]. Thus, UB chain synthesis in mitochondria sets in motion concerted adapter protein recruitment, TBK1 activation, and self-reinforcing TBK1-dependent phosphorylation of autophagy adapters to facilitate mitochondrial capture by autophagosomes [43].

The formation of a MAVS signaling complex, a mitochondria-associated adapter protein, results in the phosphorylation and nuclear translocation of IRF3/IRF7 by TBK1 and/or IKKε, as well as the activation of NF-κB to induce IFN-I and procellular cytokines. inflammatory [44,45]. Furthermore, recent studies suggest that viral infection can induce the formation of very large MAVS aggregates, which can activate IRF3 [45].

TBK1 has also been shown to be a critical and effective mechanism for regulating DRP1 function and mitochondrial dynamics [42]. In a recent study, it was shown that upon activation of RLR-MAVS signaling, TBK1 directly and massively phosphorylated DRP1 in the MAVS signaling complex upon detection of innate RNA, which deactivated it and prevented its high-order oligomerization and fragmentation function. mitochondria, eliminating the mitochondrial incision function of DRP1 [42]. Furthermore, analysis showed that the TBK1-DRP1 axis was essential for the assembly of large MAVS aggregates and healthy antiviral immunity and underlying nutrient-triggered mitochondrial dynamics and cell fate determination [42].

### 3.4. Mitochondrial Dynamics

Evidence from several studies suggests that molecular communication between innate immune signaling and mitochondrial dynamics regulates the processes of infection by microorganisms [21,22]. In particular, MFN2 appears to be a critical player in linking mitochondrial dynamics, autophagy (xenophagy and mitophagy), and innate immune responses against viruses, bacteria, and parasites, and may play an essential role in activating innate immune pathways in mitochondria-associated membranes and other specialized subcellular regions, coordinating metabolism [22].

Recent studies have shown that the AMP-activated protein kinase energy sensor interacts directly with MFN2, inducing autophagy in response to energy stress, confirming that MFN2 is essential for immune system activation during bacterial infection [22]. In addition, MFN2 is required for mitochondrial respiration and ROS production, in which it promotes cytokine and nitric oxide secretion, induces autophagy and apoptosis, and enhances antigen processing [22].

Mitochondrial dynamics also serve as a signaling platform and activation of the NLRP3 inflammasome on macrophages, altering mtROS, lipids, and membrane potential [46,47]. MFN2 plays an important role in NLRP3 inflammasome activation in bone marrow-derived macrophages after RNA virus infection and promotes association between NLRP3 and mitochondrial antiviral signaling protein (MAVS), which is followed by mitochondrial localization of NLRP3 and inflammasome activation [47]. Furthermore, mitochondrial fragmentation, by MFN2 depletion, reduces NLRP3 activity, and DRP1 ablation can increase or decrease NLRP3 activity, depending on its initial stimulus [46].

## 4. Mycobacteria vs. Mitochondria

Mycobacteria are one of the most important and well-adapted groups of human pathogens. The outcome of mycobacterial infections depends on their interaction with the host’s immune system, which can result in infection control, establishment of latent infection, or development of disease [9]. Among the diseases caused by mycobacteria, tuberculosis, caused by *Mycobacterium tuberculosis* (*Mtb*), is one of the oldest recorded human diseases and one of the greatest causes of morbidity and mortality in the world [7,9].

In addition to tuberculosis, leprosy, caused by *Mycobacterium leprae* (*Mlep*), is the second most common disease caused by mycobacteria in humans and, when compared to *Mtb*, presents an evolution in its genome that is extremely eroded, which took almost half of the functional genes (especially in metabolic pathways) to undergo inactivation or pseudogenation [8,9].

### 4.1. Mycobacterium tuberculosis

In this sense, *Mtb* is an intracellular bacterial pathogen that survives and replicates in macrophages, permeabilizing or destabilizing their phagosome, accessing the macrophage cytosol, and triggering a variety of pathways downstream of the cytosolic pattern recognition receptors [36].

Among its targets, *Mtb* targets mitochondria, like other intracellular pathogens, as these organelles are the center of cell signaling pathways and connect their metabolism to meet their nutrient demands [14]. During this process, *Mtb* alters mitochondrial structure and function for its survival, and mitochondrial alterations reported in the literature include those as part of host defense (early stage) or as in pathogenic manipulation (late stage), facilitating the survival and spread of the pathogen [14].

Among the immunological pathways that mitochondria act in mycobacterial infection, studies report that both *Mtb* genomic DNA and host mitochondrial DNA bind to cGAS cyclic GMP-AMP synthesis to activate the STING-TBK1-IRF3 pathway in response to different strains of *M. tuberculosis*, such as Erdman and CDC1551 [29,39]. The mycobacterial cyclic dinucleotide c-di-AMP, a key pathogen-associated molecular pattern, also triggers STING signaling in host cells, inducing IFN-I responses [39]. Furthermore, inflammatory pathways such as IFN-I expression, ubiquitin-mediated selective autophagy, and inflammasome activation/programmed cell death, are activated by damaged mitochondria when they release ligands such as mtDNA into the cytosol [36]. During *Mtb* infection, ex vivo genetic studies have proposed that most of the IFN-I expression occurs downstream of cGAS, as the response is completely disrupted in Cgas2/2 murine and human macrophage cell lines infected with *Mtb* and primary murine macrophages [36].

Another important factor is the influence of mtROS on the activation of host-induced apoptosis, which is also closely associated with mitochondrial bioenergetics [14]. mtROS are channeled to phagosomes of the surrounding mitochondrial cluster, and thus, the resident *Mtb* is exposed to mtROS. Excess mtROS signals to the TNF-α-dependent extrinsic apoptosis pathway and mtROS also lead to the mitochondrial membrane permeability transition and Cytochrome C release and, therefore, activate the intrinsic apoptosis pathway, being a known *Mtb* factor for inducing mtROS or *Mtb*-TNT [14].

Inflammasome activation also plays an important role in the immune response against *Mtb*, as deletion of key inflammasome factors, including IL-18, IL-1b, and IL-1 receptor (IL-1R), confers susceptibility to *Mtb*, in addition to cellular stress or mitochondrial dysfunction that causes release of mito-DAMPs, including mtROS, cardiolipin, and extracellular ATP that can activate the NLRP3 inflammasome, although certain aspects of the molecular mechanisms that drive inflammasome activation and the ability of *Mtb* to manipulate this pathway inside the infected macrophages remain obscure [35,36,37].

Of note, while the nature of these additional sensors remains elusive, several in vivo studies suggest that TLR9 contributes to host resistance to *Mtb*, and RNA detection pathways have been shown to contribute to IFN-I responses [36].

It is important to report that the effect of mitochondrial dynamics in the pathogenesis of tuberculosis is a factor still little explored, although there are studies that have shown that *M. tuberculosis* induces changes in mitochondrial membrane potential, cytochrome c release and modification of mitochondrial dynamics [21,48]. Furthermore, mitochondrial dynamics, including fission and fusion, play a critical role in maintaining mitochondria for ATP production, which is directly linked to host defense against *Mycobacterium tuberculosis* infection [48,49].

In this sense, MFN2 has been shown to be implicated in controlling the ability of *Mtb* to survive and replicate within macrophages ex vivo and in a mouse infection model, consistent with GWAS that identified human SNPs in MFN2 associated with tuberculosis susceptibility in a Han Chinese population [36]. It was also reported in another study that MFN2 expression was reduced after infection of the H37Rv strain (pathogenic *M. tuberculosis* laboratory strain) in human monocytic THP-1 cells, which correlated with the structural alteration observed in the mitochondrial population [21]. Furthermore, it was observed that the mitochondrial membrane potential was significantly increased after H37Rv infection when compared to THP-1 cells infected by H37Ra (avirulent strain), also increasing mitochondrial ETC activity in host cells, while H37Ra inhibited the ETC [21].

Mitochondrial fusion mediated by MFN1 has also been shown to be essential for the mycobactericidal activity of macrophages through the regulation of ATP-dependent autophagy, and the metabolism pathway mediated by this mitofusin is a potential target for the development of therapeutic strategies against tuberculosis [49].

In studies investigating *Mtb* virulence factors, factor Rv1411c (LprG/p27) has been shown to induce mitochondrial fission and reduce cellular respiratory rate and modify the kinetics of host mitochondrial Ca2+ uptake in response to agonist stimulation [14,48]. On the other hand, virulence factor Rv1818c (PE_PGRS33) promoted mitochondrial fusion with no effect on Ca2+ uptake and cell respiration rate, which suggests that two different virulence factors of the same pathogen induce differential effects on mitochondrial dynamics, cellular respiration, and mitochondrial uptake of Ca2+ [14,48].

### 4.2. Mycobacterium leprae

The pathogen *M. leprae* is an obligate intracellular parasite and mainly affects the skin and peripheral nerves [8]. During evolutionary erosion, *Mlep* developed a dependence on the host’s energy production and nutritional products, and as a result, parasitic life and adaptation may have shaped the host’s genetic susceptibility to leprosy [4,8]. *Mlep* and *Mtb* are highly related pathogens, and the severity of leprosy and tuberculosis is highly correlated with IFN-I responses in vivo [36]. Numerous susceptibility *loci* have been associated with exacerbated leprosy in human patients, most of them in immune sensors and signaling molecules [36].

Recent studies have shown that mutations in leprosy-associated genes may play important roles in *M. leprae* infection, pointing to a role of mitochondria in regulating immunological outcomes during mycobacterial infection [8,36].

One of the most studied genes associated with susceptibility to leprosy and other inflammatory diseases such as Crohn’s disease is *LRRK2*, which encodes a massive, multifunctional protein with GTPase and kinase activity that contributes to mitochondrial homeostasis, lysosomal acidification, and autophagy/mitophagy [36,50,51]. *LRRK2* was identified by a genome-wide association study (GWAS) as one of the leprosy susceptibility genes in the Han Chinese population [8,51].

It is noteworthy that a variety of genes involved in the pathogenesis of leprosy have been observed, such as *PARK2/PACRG* and *TNFSF15*, participating in the regulation of host cell apoptosis, and genes that participate in the formation and maintenance of granulomas, such as *TNF*, *LTA,* and *IFNG* [50].

Variants in genes such as *PARK2*, *PARL* (rhomboid-like protein associated with presenilins), and *PINK1* (PTEN-induced kinase 1) are critical for mitochondrial turnover through mitophagy and increase the risk of developing leprosy [36]. PINK1 is a serine/threonine kinase protein located in mitochondria that can phosphorylate Parkin, leading to activation of Parkin E3-ubiquitin ligase (PARK2) and the NF-κ B signaling pathway [8,36]. Parkin-mediated ubiquitination of mitochondrial substrates, including BCL2 (B 2 cell lymphoma), mitofusins, and VDAC (voltage-gated anion channel), provides the signal that recruits autophagy adapters that bind tagged/damaged mitochondria to autophagosomes [36].

The *OPA1* gene, which encodes a mitochondrial inner membrane protein, has been associated with leprosy susceptibility possibly by affecting mitochondrial function and antimicrobial pathways, and studies identified two *OPA1* variants that showed a positive association with increased risk for leprosy (rs414237), where it correlated with lower *OPA1* mRNA expression, suggesting that the susceptibility phenotype is attributable to the loss of *OPA1* [8,36]. *PARL* can interact with *OPA1* during apoptosis, regulating apoptotic crest remodeling and cytochrome c release [8]. Furthermore, *PARL* together with *OPA1* can control mitochondrial morphology and participate in mitochondrial adaptation to heat shock [8].

It is important to emphasize that, in relation to advances in studies on the influence of mitochondrial dynamics in the pathogenesis of leprosy, there are no data in the scientific literature published so far on this topic.

Regardless, mutations in factors necessary to replicate and maintain the mitochondrial genome, with mutations in *TFAM*, the mitochondrial transcription factor A, and *POLG*, the mitochondrial DNA polymerase, are also associated with mycobacterial susceptibility and leprosy [36]. Another factor that has been suggested to influence the leprosy susceptibility process is genomic ancestry [52]. Notably, among the forms of genomic ancestry is mitochondrial ancestry through mitochondrial haplogroups.

## 5. Mitochondrial Haplogroups

The analysis of mitochondrial haplogroups of a particular lineage is of great importance in scientific research and diagnosis [53]. During human evolution, different populations have acquired mtDNA polymorphisms that characterize genetic haplotypes inherited from the mother [31,53].

Due to population migration, distinct mitochondrial haplogroups are associated with different continental ancestors, including Africans, Europeans, Native Americans, Asians, and Oceanians, allowing an accurate classification of maternal genetic ancestry in large datasets using a small subset of mitochondrial markers [54].

In this sense, the characterization of mitochondrial haplogroups allowed both the tracking of global migrations of human populations and the detection of some mitochondrial diseases, observed more frequently in specific haplogroups, in addition to complementing previous knowledge about the biological composition of this population, which make this approach useful as screening methods in medical genetics [31,53,55].

The mtDNA analysis for ancestry testing purposes is performed using a specific large region of the mitochondrial genome [56]. The NCR, the control region of the mtDNA molecule, is the most informative in terms of maternal ancestry and has three hypervariable regions (HVR-I, HVR-II, and HVR-III), which collectively can form ancestral haplogroups through the presence of specific motifs [55].

Due to the specific characteristics of mtDNA, such as its maternal inheritance, absence of recombination, and high mutation rate, each mtDNA lineage has accumulated mutations during evolution, and a group that share certain ancestral mtDNA variants may belong to distinguishable haplogroups [4].

In this perspective, several studies demonstrated that mitochondrial ancestry affected genetic susceptibility to many diseases, including maternally inherited diseases, e.g., Leber hereditary optic neuropathy (LHON) and myoclonic epilepsy with irregular red fibers (MERRF), metabolic diseases, infectious diseases, acquired immunodeficiency syndrome (AIDS), and sepsis [4]. However, whether mitochondrial haplogroups can confer a genetic susceptibility to mycobacterial infection is an interesting and important question, and there are still few studies, with one of the only studies showing that in the Han Chinese population haplogroups did not confer susceptibility to leprosy [4]. Thus, further studies in different populations are needed to better clarify the possible role of mitochondrial ancestry in the development of leprosy.

## 6. Nuclear and Mitochondrial Ancestry

Genetic ancestry has long been recognized as a key topic in population genetics and has been shown to be an influential factor in the development of omics studies, including genome-wide association studies (GWAS), epigenetic studies, and proteomics studies [57]. In this sense, GWAS are often performed on populations of unrelated individuals to identify susceptibility *loci* for complex human characteristics, such as infectious and parasitic diseases [58].

Among these genetic analyses, mitochondrial genome association studies (MiWAS) stand out, in which single nucleotide mitochondrial polymorphisms (mtSNPs) that are associated with diseases or related phenotypes are identified [59]. Variation in these genes can alter mitochondrial function and increase the risk of certain diseases in specific ethnicities, as mtDNA reflects historical patterns of human migration [59].

## 7. Conclusions

Mitochondria play an essential role in regulating comprehensive functions in humans, both at the cellular and systemic level, and are often targets for many pathogenic viruses and intracellular bacteria [7,14,27]. This regulation extends far beyond simply bioenergetic generation and includes, but is not limited to, the precise and differentiated control of innate immune response activation and signaling [27].

It is noteworthy that several bacterial pathogens produce pathogenicity factors that participate in the modulation of mitochondrial functions that decide the fate of the host cell [7]. In addition, this organelle represents a rich source of DAMPs that can potently trigger the innate immune system, such as ATP, ROS production, inflammasomes, and mtDNA which, due to its bacterial origin, is particularly effective in initiating inflammatory and antiviral signaling [26,29].

Mycobacterial infection and its outcome depend on the complex interaction between the intracellular pathogen and the host’s immune system [7,9]. Through this interaction, mycobacteria have evolved to adapt to their host, developing intricate mechanisms to subvert the immune response [9]. However, there is still a lack of studies associating mycobacterial infection with mitochondria in the global literature.

Therefore, advancing the understanding of immunological pathways in which mitochondria act, their relationship with haplogroups, and the precise mechanisms by which mycobacteria interact with important host organelles to affect their function will provide a better understanding of the pathogenesis of this parasitic agent and reveal additional new pathways through which these systems are intertwined.

## Data Availability

Not applicable.

## References

[1] Cavalcante G.C., Marinho A.N.R., Anaissi A.K., Vinasco-Sandoval T., Ribeiro-dos-Santos A., Vidal A.F., de Araújo G.S., Demachki S., Ribeiro-dos-Santos Â. (2019). Whole Mitochondrial Genome Sequencing Highlights Mitochondrial Impact in Gastric Cancer. Sci. Rep..

[2] Yan C., Duanmu X., Zeng L., Liu B., Song Z. (2019). Mitochondrial DNA: Distribution, Mutations, and Elimination. Cells.

[3] Oliveira M.F., Medeiros R.C.A., Mietto B.S., Calvo T.L., Mendonça A.P.M., Rosa T.L.S.A., da Silva D.S., Vasconcelos K.G.D.C.D., Pereira A.M.R., de Macedo C.S. (2021). Reduction of Host Cell Mitochondrial Activity as Mycobacterium Leprae’s Strategy to Evade Host Innate Immunity. Immunol. Rev..

[4] Wang D., Su L.Y., Zhang A.M., Li Y.Y., Li X.A., Chen L.L., Long H., Yao Y.G. (2012). Mitochondrial DNA Copy Number, but Not Haplogroup, Confers a Genetic Susceptibility to Leprosy in Han Chinese from Southwest China. PLoS ONE.

[5] White C., Franco-Paredes C. (2015). Leprosy in the 21st Century. Clin. Microbiol. Rev..

[6] Salgado C.G., Pinto P., Bouth R.C., Gobbo A.R., Messias A.C.C., Sandoval T.V., dos Santos A.M.R., Moreira F.C., Vidal A.F., Goulart L.R. (2018). MiRNome Expression Analysis Reveals New Players on Leprosy Immune Physiopathology. Front. Immunol..

[7] Dubey R.K. (2016). Assuming the role of mitochondria in mycobacterial infection. Int. J. Microbiol..

[8] Wang D., Zhang D.F., Feng J.Q., Li G.D., Li X.A., Yu X.F., Long H., Li Y.Y., Yao Y.G. (2016). Common Variants in the PARL and PINK1 Genes Increase the Risk to Leprosy in Han Chinese from South China. Sci. Rep..

[9] Fraga A.G., Barbosa A.M., Ferreira C.M., Fevereiro J., Pedrosa J., Torrado E. (2018). Immune-Evasion Strategies of Mycobacteria and Their Implications for the Protective Immune Response. Curr. Issues Mol. Biol..

[10] MacNeil A., Glaziou P., Sismanidis C., Date A., Maloney S., Floyd K. (2020). Global Epidemiology of Tuberculosis and Progress Toward Meeting Global Targets—Worldwide, 2018. Morb. Mortal. Wkly. Rep..

[11] Fukunaga R., Glaziou P., Harris J.B., Date A., Floyd K., Kasaeva T. (2021). Epidemiology of Tuberculosis and Progress Toward Meeting Global Targets—Worldwide, 2019. Morb. Mortal. Wkly. Rep..

[12] Sarode G., Sarode S., Anand R., Patil S., Jafer M., Baeshen H., Awan K.H. (2020). Epidemiological Aspects of Leprosy. Dis. Month.

[13] Miguel C.B., da Mota P.B., Afonso B.O., Agostinho F., Cazzaniga R.A., de Abreu M.C.M., Oliveira C.J.F., Rodrigues W.F. (2021). Leprosy Morbidity and Mortality in Brazil: 2008–2018. Braz. J. Infect. Dis..

[14] Mohareer K., Medikonda J., Vadankula G.R., Banerjee S. (2020). Mycobacterial Control of Host Mitochondria: Bioenergetic and Metabolic Changes Shaping Cell Fate and Infection Outcome. Front. Cell. Infect. Microbiol..

[15] Nguyen N.N.Y., Kim S.S., Jo Y.H. (2020). Deregulated Mitochondrial DNA in Diseases. DNA Cell Biol..

[16] Cavalcante G.C., Magalhães L., Ribeiro-Dos-Santos Â., Vidal A.F. (2020). Mitochondrial Epigenetics: Non-Coding RNAs as a Novel Layer of Complexity. Int. J. Mol. Sci..

[17] Roger A.J., Muñoz-Gómez S.A., Kamikawa R. (2017). The Origin and Diversification of Mitochondria. Curr. Biol..

[18] Chapman J., Ng Y.S., Nicholls T.J. (2020). The Maintenance of Mitochondrial DNA Integrity and Dynamics by Mitochondrial Membranes. Life.

[19] Andrieux P., Chevillard C., Cunha-Neto E., Nunes J.P.S. (2021). Mitochondria as a Cellular Hub in Infection and Inflammation. Int. J. Mol. Sci..

[20] Tilokani L., Nagashima S., Paupe V., Prudent J. (2018). Mitochondrial Dynamics: Overview of Molecular Mechanisms. Essays Biochem..

[21] Khan S., Raj D., Jaiswal K., Lahiri A. (2020). Modulation of Host Mitochondrial Dynamics during Bacterial Infection. Mitochondrion.

[22] Kim I.S., Silwal P., Jo E.K. (2021). Mitofusin 2, a Key Coordinator between Mitochondrial Dynamics and Innate Immunity. Virulence.

[23] Tiku V., Tan M.W., Dikic I. (2020). Mitochondrial Functions in Infection and Immunity. Trends Cell Biol..

[24] Bahat A., MacVicar T., Langer T. (2021). Metabolism and Innate Immunity Meet at the Mitochondria. Front. Cell Dev. Biol..

[25] Chowdhury A., Witte S., Aich A. (2022). Role of Mitochondrial Nucleic Acid Sensing Pathways in Health and Patho-Physiology. Front. Cell Dev. Biol..

[26] Weinberg S.E., Sena L.A., Chandel N.S. (2015). Mitochondria in the Regulation of Innate and Adaptive Immunity. Immunity.

[27] Banoth B., Cassel S.L. (2018). Mitochondria in Innate Immune Signaling. Transl. Res. J. Lab. Clin. Med..

[28] Faas M.M., de Vos P. (2020). Mitochondrial Function in Immune Cells in Health and Disease. Biochim. Biophys. Acta Mol. Basis Dis..

[29] Riley J.S., Tait S.W. (2020). Mitochondrial DNA in Inflammation and Immunity. EMBO Rep..

[30] Harrington J.S., Choi A.M.K., Nakahira K. (2017). Mitochondrial DNA in Sepsis. Curr. Opin. Crit. Care.

[31] Zhang Q., Raoof M., Chen Y., Sumi Y., Sursal T., Junger W., Brohi K., Itagaki K., Hauser C.J. (2010). Circulating Mitochondrial DAMPs Cause Inflammatory Responses to Injury. Nature.

[32] Liu Y., Yan W., Tohme S., Chen M., Fu Y., Tian D., Lotze M., Tang D., Tsung A. (2015). Hypoxia Induced HMGB1 and Mitochondrial DNA Interactions Mediate Tumor Growth in Hepatocellular Carcinoma through Toll Like Receptor 9. J. Hepatol..

[33] West A.P., Shadel G.S. (2017). Mitochondrial DNA in Innate Immune Responses and Inflammatory Pathology. Nat. Rev. Immunol..

[34] Picca A., Calvani R., Coelho-Junior H.J., Marzetti E. (2021). Cell Death and Inflammation: The Role of Mitochondria in Health and Disease. Cells.

[35] Zhong Z., Umemura A., Sanchez-Lopez E., Liang S., Shalapour S., Wong J., He F., Boassa D., Perkins G., Ali S.R. (2016). NF-ΚB Restricts Inflammasome Activation via Elimination of Damaged Mitochondria. Cell.

[36] Patrick K.L., Watson R.O. (2021). Mitochondria: Powering the Innate Immune Response to Mycobacterium Tuberculosis Infection. Infect. Immun..

[37] Zhong Z., Liang S., Sanchez-Lopez E., He F., Shalapour S., Lin X.-J., Wong J., Ding S., Seki E., Schnabl B. (2018). New Mitochondrial DNA Synthesis Enables NLRP3 Inflammasome Activation. Nature.

[38] Sandhir R., Halder A., Sunkaria A. (2017). Mitochondria as a centrally positioned hub in the innate immune response. Biochim. Biophys. Acta (BBA)-Mol. Basis Dis..

[39] Sun Y., Zhang W., Dong C., Xiong S. (2020). Mycobacterium Tuberculosis MmsA (Rv0753c) Interacts with STING and Blunts the Type I Interferon Response. mBio.

[40] Hopfner K.P., Hornung V. (2020). Molecular Mechanisms and Cellular Functions of CGAS–STING Signalling. Nat. Rev. Mol. Cell Biol..

[41] Nozawa T., Sano S., Minowa-Nozawa A., Toh H., Nakajima S., Murase K., Aikawa C., Nakagawa I. (2020). TBC1D9 Regulates TBK1 Activation through Ca2+ Signaling in Selective Autophagy. Nat. Commun..

[42] Chen S., Liu S., Wang J., Wu Q., Wang A., Guan H., Zhang Q., Zhang D., Wang X., Song H. (2020). TBK1-Mediated DRP1 Targeting Confers Nucleic Acid Sensing to Reprogram Mitochondrial Dynamics and Physiology. Mol. Cell.

[43] Heo J.M., Ordureau A., Paulo J.A., Rinehart J., Harper J.W. (2015). The PINK1-PARKIN Mitochondrial Ubiquitylation Pathway Drives a Program of OPTN/NDP52 Recruitment and TBK1 Activation to Promote Mitophagy. Mol. Cell.

[44] Jacobs J.L., Coyne C.B. (2013). Mechanisms of MAVS Regulation at the Mitochondrial Membrane. J. Mol. Biol..

[45] Cui J., Chen Y., Wang H.Y., Wang R.-F. (2014). Human Vaccines & Immunotherapeutics Mechanisms and Pathways of Innate Immune Activation and Regulation in Health and Cancer. Hum. Vaccines Immunother..

[46] Rambold A.S., Pearce E.L. (2018). Mitochondrial Dynamics at the Interface of Immune Cell Metabolism and Function. Trends Immunol..

[47] Xie J.H., Li Y.Y., Jin J. (2020). The Essential Functions of Mitochondrial Dynamics in Immune Cells. Cell. Mol. Immunol..

[48] Aguilar-López B.A., Correa F., Moreno-Altamirano M.M.B., Espitia C., Hernández-Longoria R., Oliva-Ramírez J., Padierna-Olivos J., Sánchez-García F.J. (2019). LprG and PE_PGRS33 Mycobacterium Tuberculosis Virulence Factors Induce Differential Mitochondrial Dynamics in Macrophages. Scand. J. Immunol..

[49] Ning Y., Cai Y., Dai Y., Li F., Mo S., Werz O., Chen X. (2021). Mitochondrial Fusion Mediated by Mitofusin 1 Regulates Macrophage Mycobactericidal Activity by Enhancing Autophagy. Infect. Immun..

[50] Guerreiro L.T.A., Robottom-Ferreira A.B., Ribeiro-Alves M., Toledo-Pinto T.G., Brito T.R., Rosa P.S., Sandoval F.G., Jardim M.R., Antunes S.G., Shannon E.J. (2013). Gene Expression Profiling Specifies Chemokine, Mitochondrial and Lipid Metabolism Signatures in Leprosy. PLoS ONE.

[51] Wang D., Xu L., Lv L., Su L.Y., Fan Y., Zhang D.F., Bi R., Yu D., Zhang W., Li X.A. (2015). Association of the LRRK2 Genetic Polymorphisms with Leprosy in Han Chinese from Southwest China. Genes Immun..

[52] Pinto P., Salgado C., Santos N.P.C., Santos S., Ribeiro-dos-Santos Â. (2015). Influence of Genetic Ancestry on INDEL Markers of NFKβ1, CASP8, PAR1, IL4 and CYP19A1 Genes in Leprosy Patients. PLoS Negl. Trop. Dis..

[53] Dür A., Huber N., Parson W. (2021). Fine-Tuning Phylogenetic Alignment and Haplogrouping of MtDNA Sequences. Int. J. Mol. Sci..

[54] Smieszek S., Mitchell S.L., Farber-Eger E.H., Veatch O.J., Wheeler N.R., Goodloe R.J., Wells Q.S., Murdock D.G., Crawford D.C. (2018). Hi-MC: A Novel Method for High-Throughput Mitochondrial Haplogroup Classification. PeerJ.

[55] Schaan A.P., Costa L., Santos D., Modesto A., Amador M., Lopes C., Rabenhorst S.H., Montenegro R., Souza B.D.A., Lopes T. (2017). MtDNA Structure: The Women Who Formed the Brazilian Northeast. BMC Evol. Biol..

[56] Kirkpatrick B.E., Rashkin M.D. (2017). Ancestry Testing and the Practice of Genetic Counseling. J. Genet. Couns..

[57] Yang H.C., Chen C.W., Lin Y.T., Chu S.K. (2021). Genetic Ancestry Plays a Central Role in Population Pharmacogenomics. Commun. Biol..

[58] Santos N.P.C., Ribeiro-Rodrigues E.M., Ribeiro-dos-Santos Â.K.C., Pereira R., Gusmão L., Amorim A., Guerreiro J.F., Zago M.A., Matte C., Hutz M.H. (2010). Assessing Individual Interethnic Admixture and Population Substructure Using a 48-Insertion-Deletion (INSEL) Ancestry-Informative Marker (AIM) Panel. Hum. Mutat..

[59] Miller B., Arpawong T., Jiao H., Kim S.-J., Yen K., Mehta H., Wan J., Carpten J., Cohen P. (2019). Comparing the Utility of Mitochondrial and Nuclear DNA to Adjust for Genetic Ancestry in Association Studies. Cells.

